# Trajectories of mental health and well-being across healthcare workers during COVID-19 pandemic: a Swiss longitudinal study

**DOI:** 10.3389/frhs.2026.1883340

**Published:** 2026-10-27

**Authors:** Chetna Bhatia, Gregory Moullec, Sandrine Corbaz-Kurth, Laure Vieux, Hannah Wozniak, Pauline Roos, Jean-Claude Suard, Lamyae Benzakour, Sara Cereghetti, Rafaël Weissbrodt, Jacques A. Pralong

**Affiliations:** 1 SwissMedPro Health Services SA, Geneva, Switzerland; 2 Universite de Montreal Ecole de Sante Publique, Montreal, Canada; 3 Hopitaux Universitaires Geneve, Geneva, Switzerland; 4 Suva, Lucerne, Switzerland; 5 HES-SO Valais-Wallis Haute Ecole de Sante, Sion, Switzerland

**Keywords:** COVID-19 pandemic, healthcare workers, mental health, prevention, resilience, well-being

## Abstract

This longitudinal study examined the trajectories of healthcare workers’ mental health from the first to the second COVID-19 wave (May 2020–December 2020) in a Swiss tertiary hospital, and their association with individual and organizational factors. For each mental health outcome (depression, anxiety, peritraumatic stress, and well-being), a separate analysis identified two trajectories: one with higher, persistent levels of symptoms and one that was resilient, with low-level symptoms. The persistent trajectory comprised about 25%–30% of participants for anxiety, depression and peritraumatic stress and 47% for well-being; because the classifications were outcome-specific, membership differed across outcomes (about 15% of participants were on a persistent trajectory for all four outcomes and 46% resilient for all four). We found that women, nurses, caregivers, non-managerial employees, and frontline COVID-19 workers were more likely to follow a persistent trajectory. Organizational risk factors were a high workload and an irregular work schedule. These results provide a basis for implementing risk-adapted prevention measures and resilience-promotion programs to protect health workers from the impact of major crises. Future research should address the needs of vulnerable health workers to provide evidence-based health promotion.

## Introduction

The COVID-19 pandemic generated significant psychological stress on global healthcare systems, presenting an unparalleled crisis. More than three years later, its repercussions are still unfolding, not only in persistent staff shortages and growing burnout rates, but also in the lingering mental health issues of frontline workers (1–3). While the healthcare organizations intend to strengthen their systems, it is essential to recognize the continuing impact of the pandemic on the mental health of healthcare workers (HCWs). Comprehending this impact is now more critical than ever.

Most existing studies have mainly focused on cross-sectional overviews of mental health during the early stages of the pandemic, documenting high levels of anxiety, depression, and burnout among HCWs (4–7). While significant, these data are unable to convey the evolution of psychological distress over the course of time. Beyond the acute phase of a crisis, our ability to recognize the prolonged pathways of vulnerability and resilience, and to develop tailored interventions, remains limited. As public health systems evolve from emergency response to long-term recovery, recognizing subgroups of HCWs who are still at risk has become critical.

A meta-analysis by Sun et al. (2023) has suggested that mental health symptoms in the general population may have gradually normalized post-pandemic (8). Recent findings indicate that healthcare workers, especially those in frontline positions or with unstable work conditions, have not experienced the same improvements in mental health as others (9, 10). For example, in Switzerland, data collection shows ongoing or worsening psychological strain in hospital employees after the initial pandemic waves. It thus indicates the importance of conducting studies that explore deeper into precise occupational patterns, especially for workers facing multiple stressors in institutional settings.

The objectives of this longitudinal study were threefold: (1) to identify the trajectories of anxiety, depression, peritraumatic distress and well-being among healthcare workers between May and December 2020; (2) to determine which sociodemographic, occupational and organizational characteristics were associated with membership to these trajectories; and (3) to characterize for each outcome, the subgroups of healthcare workers most likely to follow a persistent distress trajectory.

Our study demonstrates distinct patterns that facilitate crisis interventions in women, nurses, non-managers and frontline COVID-19 workers. Beyond describing how distress unfolded during the pandemic, our findings point to organizational and psychosocial factors associated with slower recovery. To build more resilient institutions, healthcare systems should address the organizational conditions associated with persistent distress – notably heavy workloads and atypical schedules – because, as our data show, these conditions are linked to symptoms that do not resolve spontaneously between crisis peaks.

## Materials and methods

### Design and recruitment

This prospective cohort study was conducted between May 2020 and December 2020 in a Swiss University hospital. All 13,570 employees received an email inviting them to participate in the study, explaining its purpose, methodology, and confidentiality measures at all levels of the organization. The online survey was hosted on the RedcapTM platform – a secure survey management platform and research database (11). Participants were informed and gave consent before completing the survey. Participants completed each survey through a secure individualized REDCap link. Responses were linked longitudinally using a pseudonymous study identifier while preserving participant anonymity throughout the study. To allow longitudinal linkage across waves while preserving anonymity, each participant was associated with a unique pseudonymous code that linked their responses across the three assessments without encoding any identifying information. The questionnaire was distributed three times via internal May 20 (baseline, end of first COVID-19 wave), September 20 (T1, inter-wave period), and December 20 (T2, second wave). The study protocol and data collection were approved by the Swiss ethics committee (BASEC ID 2020-00935).

### Data collection and materials

Each survey assessed four mental health indicators: anxiety, depression, peri-traumatic stress, and well-being – using validated questionnaires.

*Generalized Anxiety Disorder – 7 items (GAD-7)*: The Generalized Anxiety Disorder 7-item scale (GAD-7) is a validated instrument (12, 13) used to assess the severity of generalized anxiety disorder. Internal consistency is excellent (Cronbach's *α* = .92) and test-retest reliability is high (intraclass correlation = .83) (13). Four anxiety levels are proposed depending on the scores: no anxiety (0–4 pts), mild (5–9 pts), moderate (10–14 pts), and severe anxiety (15–21 pts) (13).

*The Patient Health Questionnaire 9-item scale (PHQ-9)* assesses the severity of depressive symptoms (14). Internal consistency has been reported as good to excellent (Cronbach's *α* = .80 to.89) across different populations (15, 16). Four depression levels are proposed according to the scores: no depression (0–4 pts), mild (5–9 pts), moderate (10–14 pts), moderately severe (15–19 pts), and severe depression (20–27 pts). The PHQ-9 has demonstrated excellent psychometric properties (5, 16).

*The Peritraumatic Distress Inventory (PDI)* assesses emotional and physiological distress experienced during or immediately after a traumatic event using 13 questions (17). In the present study it was administered identically at the three assessments and is therefore used as a repeated indicator of acute distress during the successive phases of the pandemic, rather than as a measure of the peritraumatic response to a single, dated event. This use extends beyond the conditions under which the instrument was validated – the reference event is not identical for all participants, and peritraumatic distress is a state expected to decline over time (18) – and PDI results are therefore interpreted with caution. Its psychometric properties are well-established, with internal consistency reported between *α* = .75 and *α* = .76 and good temporal stability (test–retest r = .74) (17). A PDI score of ≥ 14 indicates a risk for developing post-traumatic stress within one month of the traumatic event (17). The PDI is considered a valid and reliable instrument (17).

The WHO-5 Well-Being Index is a validated 5-item scale that measures psychological well-being (19). The raw score ranges from 0 to 25 and is multiplied by 4 to yield a 0–100 score. A score of 0 represents the poorest well-being, while 100 represents the best possible well-being. Internal consistency has been reported as acceptable to good (*α* = .83 to.84) (20). Scores below 50 are considered indicative of poor well-being. WHO-5 scores correlate negatively with measures of depression and suicidality (18). The WHO-5 has demonstrated good psychometric properties (19). In the present sample, internal consistency (Cronbach's alpha) was .89 for the GAD-7, .86 for the PHQ-9, .86 for the PDI, and .91 for the WHO-5.

The survey also included questions on psychosocial and occupational determinants that are known to influence mental outcomes. These included indicators of work organizations (type of schedule [atypical or regular], teleworking, workload variations, transfers to COVID-19 units, and changes in hospital sector during the crisis) and sociodemographic characteristics (gender, age, marital status, dependent children, hospital sector and changes during the COVID-19 pandemic, working in COVID-19 units, professional category and hierarchical status, contract type, activity rate, seniority and country of residence).

The choice of these variables was grounded in existing knowledge about mental health risk factors among healthcare workers and employees exposed to demanding work environments. Previous findings show that workload fluctuations, family responsibilities, and working in high-exposure units contribute to psychological distress and stress responses, a pattern later confirmed in COVID-19 research (e.g. 21). Similarly, characteristics such as age, gender, and workplace exposure have long been associated with differences in anxiety, depression, and post-traumatic stress in health professionals, and have also been observed during the pandemic (e.g. 22, 23). We further included job status, contract type, seniority, marital status, presence of dependent children, activity rate, and country of residence, given their potential influence on mental health.

Including these determinants enabled us to capture well-documented individual vulnerabilities and structural features of work that are theoretically and empirically linked to mental health, while accounting for sources of heterogeneity between workers experiencing different degrees of exposure and workload.

### Data analysis

To verify the presence of selective attrition that may have influenced our results, we compared the characteristics of participants who had completed at least one follow-up survey with those who had only completed the baseline. To identify distinct, homogeneous clusters of mental health and well-being outcome trajectories over three assessment time points (May 20, Sept. 20, and Dec. 20), we applied longitudinal k-means clustering (kml package (24), run separately for each outcome (GAD-7, PHQ-9, PDI, WHO-5). Given that our data primarily comprised continuous variables, the k-means method was deemed the most suitable. For each outcome, solutions from two to seven clusters were estimated with ten re-initializations, and the number of clusters was selected using the quality criteria provided by kml, primarily the Calinski-Harabasz index, examined together with the cluster trajectory plots. For all four outcomes, a two-cluster solution maximized the Calinski-Harabasz criterion and was stable across re-initializations, distinguishing a lower-symptom (“resilient”) from a higher-symptom (“persistent”) trajectory (Supplementary Figure S1). Within each trajectory group, score changes between consecutive assessments (T0-T1, T1-T2, and T0-T2) were compared using paired t-tests, Bonferroni-corrected for the three comparisons per outcome (Supplementary Table S3). Once participants were assigned to specific trajectory groups, baseline individual and organizational characteristics were used to characterize each trajectory profile and to identify potential risk or protective factors. Variables entered into multivariable analyses were selected based on established associations with mental health and COVID-19 outcomes and on their theoretical relevance as potential determinants or confounders. Those meeting this criterion and yielding a *p*-value < 0.25 in univariate analyses were retained for further modelling. Because each trajectory outcome was binary (persistent vs. resilient), associations with baseline characteristics were tested using binary logistic (binomial) regression models, run separately for each outcome, and included age, gender, professional category, hospital department's affiliation, work in a COVID-19-unit, hierarchical status, activity rate, and work schedule. All analyses were performed using R Studio software version 1.4.1717 (25).

## Results

### Sample characteristics

At baseline (T0), 3,461 questionnaires were filled out, of 13,570 sent (response rate = 25%) (Table 1 and Figure 1). Of these, 1,552 completed at least one follow-up survey (T1 or T2) (retention rate = 45%; attrition rate = 55%). Attrition rates throughout the follow-up did not appear statistically distinct in the different subgroups (Table 2). Socio-demographic participants’ characteristics followed from May 20 to Dec. 20 (*n* = 1,552) are presented in Table 2. The sample was predominantly female (75%), with the majority aged between 30 and 59 years (88%) and with over 10 years of seniority (66%). Nurses, caregivers, and care assistants made up 45% of the workforce, followed by administrative staff (20%), medical/technical therapists and social workers (13%), doctors (11%), and logistics personnel (6%). Regarding work organization, 56% of participants reported an atypical work schedule, 25% reported work overload, 16% teleworked, 10% worked in COVID-19 units, and 17% had changed hospital sector since the beginning of the pandemic. Most participants held a long-term contract (91%) and did not have a managerial role (79%).

**Table 1:** Description of participant characteristics, at recruitment (*N* = 3,461).

	Baseline (*N* = 3461)
Participant characteristics	*N* (%)
Demographics	
Age class	
18–29	402 (12)
30–39	815 (24)
40–49	1032 (30)
50–59	1049 (30)
60+	163 (5)
Gender	
Man	897 (26)
Woman	2561 (74)
Missing	3 (0)
Marital status	
In a relationship/Married	2215 (64)
Single	767 (22)
Separated	451 (13)
Widowed	27 (1)
Missing	1 (0)
Children	
Yes	1635 (47)
No	1826 (53)
Country	
Switzerland	1927 (56)
France	1516 (44)
Other	13 (0)
Missing	5 (0)
Organizational	
Change of hospital sector since the pandemic	
Yes	594 (17)
No	2866 (83)
Missing	1 (0)
COVID unit	
Yes	330 (10)
No	3079 (89)
Don't know	50 (1)
Missing	2 (0)
Work contract	
Long-term contract	3141 (91)
Fixed-term contract	280 (8)
Student trainee	38 (1)
Hierarchical status	
High manager	277 (8)
Middle manager	422 (12)
No managerial function	2751 (79)
Missing	11 (0)
Practitioner trainer	
Yes	461 (13)
No	2997 (87)
Missing	3 (0)
Atypical work schedule	
Yes	1949 (56)
No	1507 (44)
Missing	3 (0)
Teleworking	
Yes	546 (16)
No	2907 (84)
Missing	8 (0)
Workload	
Idem	2067 (60)
Overload	875 (25)
Underload	512 (15)
Missing	7 (0)
Seniority	
0–1.9	253 (7)
2–4.9	397 (11)
5–9.9	575 (17)
10–19.9	1038 (30)
20+	1076 (31)
Missing	122 (4)
Professional categories	
Nurse/caregivers	1602 (46)
Administrative Staff	637 (18)
Physician	438 (13)
Medical-technical staff	415 (12)
Logistics staff	192 (6)
Others	176 (5)
Missing	1 (0)
Hospital sector	
Surgery	242 (7)
Intensive care	352 (9)
Primary care	194 (5)
Medical specialty	329 (9)
Women and children	428 (11)
Diagnostic (radio, labs, pathology)	209 (6)
Neurosciences	168 (4)
Oncology	76 (2)
Mental health and psychiatry	293 (7)
Readaptation and geriatric	366 (10)
Direction and common service	353 (9)
Exploitation	188 (5)
Other	263 (7)

**Figure 1:**
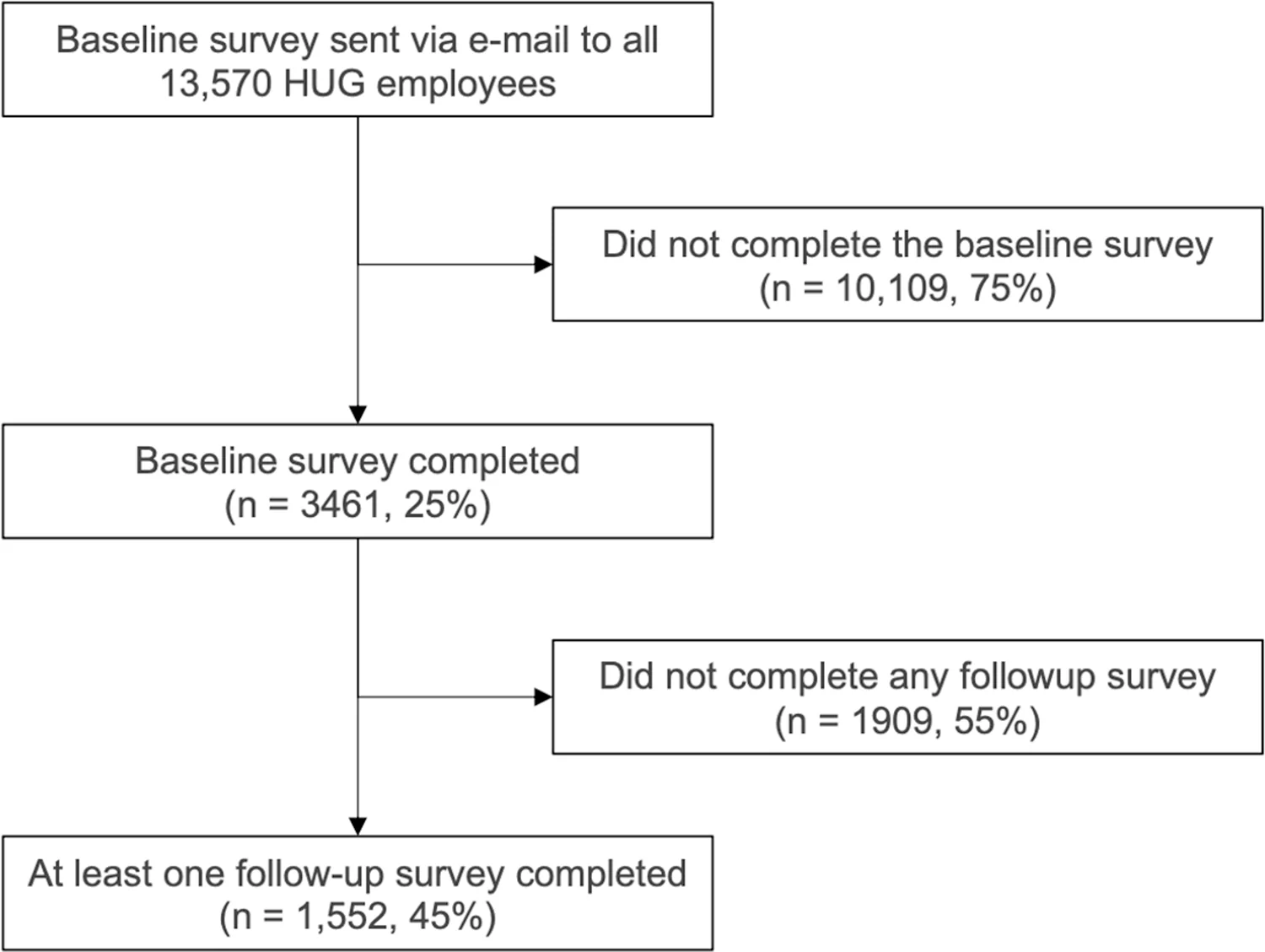
Flow chart of participants.

**Table 2:** Attrition in the cohort and comparison of participants enrolling or not in the follow-ups according to demographics and organizational characteristics (*N* = 3,461).

	Participated in follow-ups (*N* = 1,552)	Participated in baseline only (*N* = 1,909)	
Cohort attrition	*N* (%)	*N* (%)	*p-value*
Demographics			
Age class			<.001
18–29	116 (7)	286 (15)	
30–39	315 (20)	500 (26)	
40–49	522 (34)	510 (27)	
50–59	531 (34)	518 (27)	
60+	68 (4)	95 (5)	
Gender			0.16
Man	384 (25)	513 (27)	
Woman	1165 (75)	1396 (73)	
Missing	3 (0)	0 (0)	
Marital status			<.001
In a relationship/Married	1028 (66)	1187 (62)	
Single	295 (19)	472 (25)	
Separated	222 (14)	229 (12)	
Widowed	7 (0)	20 (1)	
Missing	0 (0)	1 (0)	
Children			0.005
Yes	774 (50)	861 (45)	
No	778 (50)	1048 (55)	
Country			0.51
Switzerland	874 (56)	1053 (55)	
France	674 (43)	842 (44)	
Other	4 (0)	9 (0)	
Missing	0 (0)	5 (0)	
Organizational			
Change of hospital sector since the pandemic			0.15
Yes	282 (18)	312 (16)	
No	1269 (82)	1597 (84)	
Missing	1 (0)	0 (0)	
COVID unit			0.35
Yes	137 (9)	193 (10)	
No	1390 (90)	1689 (88)	
Don't know	25 (2)	25 (1)	
Missing	0 (0)	2 (0)	
Work contract			<.001
Long-term contract	1441 (93)	1700 (89)	
Fixed-term contract	102 (7)	178 (9)	
Student trainee	9 (1)	29 (2)	
Hierarchical status			<.001
Senior manager	140 (9)	137 (7)	
Middle manager	222 (14)	200 (11)	
No manager	1188 (77)	1563 (82)	
Missing	2 (0)	9 (0)	
Practitioner trainer			<.001
Yes	234 (15)	1681 (88)	
No	1316 (85)	227 (12)	
Missing	2 (0)	1 (0)	
Atypical work schedule			0.09
Yes	899 (58)	1050 (55)	
No	652 (42)	855 (45)	
Missing	1 (0)	4 (0)	
Teleworking			0.002
Yes	279 (18)	267 (14)	
No	1272 (82)	1635 (86)	
Missing	1 (0)	7 (0)	
Workload			0.97
Idem	923 (59)	1144 (60)	
Overload	393 (26)	477 (25)	
Underload	230 (15)	282 (15)	
Missing	1 (0)	6 (0)	
Seniority			<.001
0–1.9	100 (6)	153 (8)	
2–4.9	138 (9)	259 (14)	
5–9.9	242 (16)	333 (17)	
10–19.9	482 (31)	556 (29)	
20+	547 (35)	529 (28)	
Missing	43 (3)	79 (4)	
Professional categories 1			0.08
Nurse-caregivers	700 (45)	902 (47)	
Administrative Staff	307 (20)	330 (17)	
Physician	177 (11)	261 (14)	
Medical-technical staff	200 (13)	215 (11)	
Logistics staff	91 (6)	101 (5)	
Others	77 (5)	99 (5)	
Missing	0 (0)	1 (0)	
Hospital sector			0.002
Surgery	95 (6)	147 (8)	
Intensive care	162 (10)	190 (10)	
Primary care	90 (6)	104 (5)	
Medical specialty	145 (9)	184 (10)	
Women and children	155 (10)	273 (14)	
Diagnostic (radio, labs, pathology)	95 (6)	114 (6)	
Readaptation and geriatric	184 (12)	182 (10)	
Neurosciences	74 (5)	94 (5)	
Oncology	31 (2)	45 (2)	
Mental health and psychiatry	123 (8)	170 (9)	
Direction and common service	187 (12)	166 (9)	
Exploitation	90 (6)	98 (5)	
Other	121 (8)	142 (7)	

### Mental health and well-being trajectories

We recognized two distinct trajectory groups for each mental health outcome, labeled ‘resilient’ (lower-level symptoms) and ‘persistent’ (higher-level symptoms) (Figure 2). For anxiety (GAD-7), depression (PHQ-9), and peritraumatic scores (PDI), 30%, 28%, and 25% of participants, respectively, were classified in the persistent trajectory throughout the follow-up, and 47% showed consistently lower well-being (WHO-5). Because the analyses were run separately for each outcome, trajectory membership was outcome-specific: among the 1,527 participants classified on all four outcomes, 46.2% followed a resilient trajectory on every outcome and 14.7% a persistent trajectory on every outcome, while the remaining 39.1% were persistent on one to three outcomes only. Agreement among the three symptom scales was moderate to substantial (Cohen's *κ* = 0.56–0.61), whereas well-being (WHO-5) defined a broader persistent group with weaker agreement with the symptom scales (*κ* = 0.42–0.49); most workers on a persistent symptom trajectory also reported persistently low well-being (85%–90%), but the converse was less frequent (46%–53%). Concordance details are given in Supplementary Table S4.

**Figure 2:**
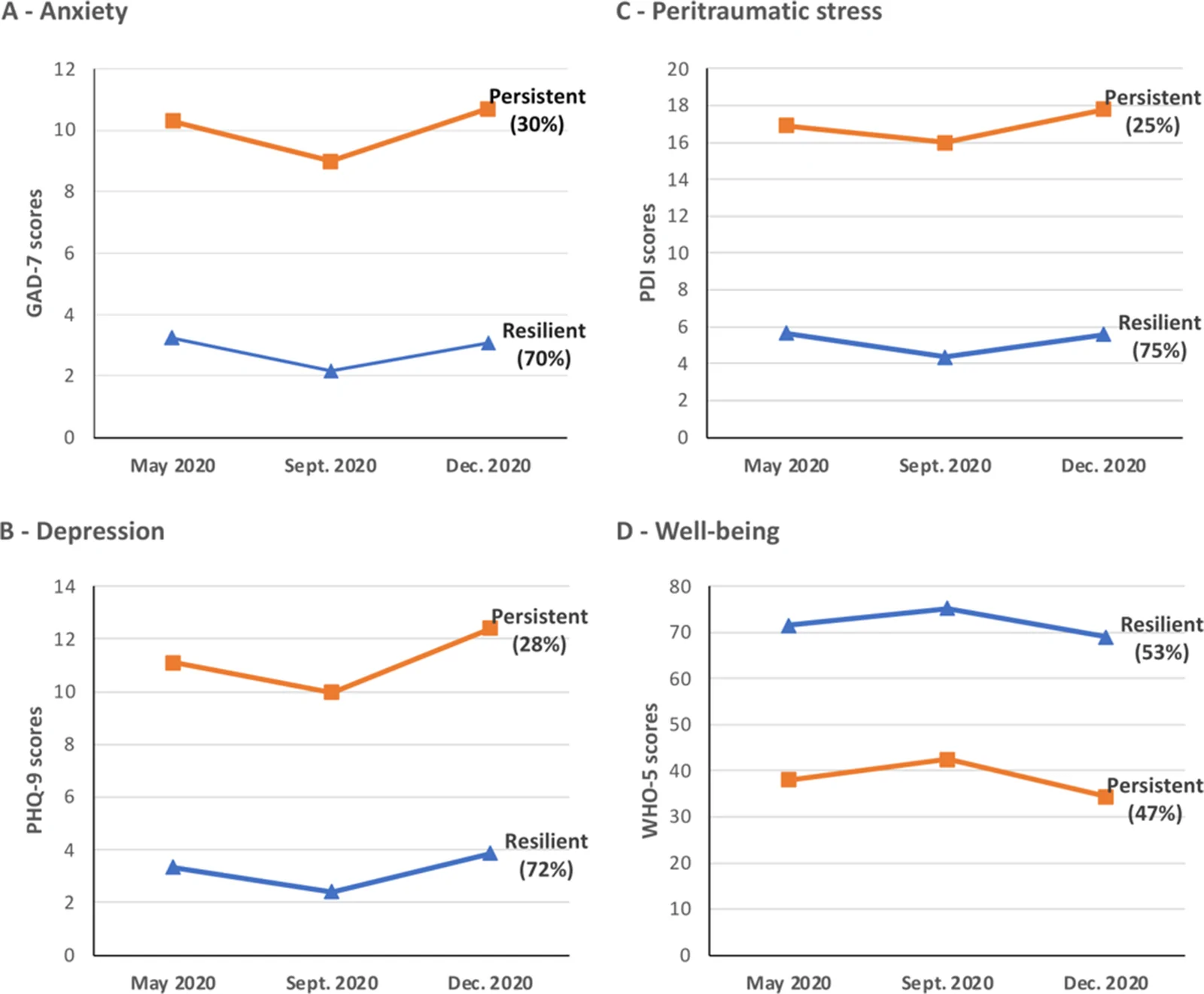
Group trajectories of mental health and well being outcomes (*N*= 1,552): **(A)** anxiety (GAD-7), **(B)** depression (PHQ-9), **(C)** peritraumatic distress (PDI), and **(D)** well-being (WHO-5) across May 2020, September 2020, and December 2020.

Mental health symptoms followed a U-shaped pattern, with higher scores observed during the first and second COVID-19 peaks, and a temporary improvement in between. Within both trajectory groups, the improvement between T0 and T1 and the subsequent worsening between T1 and T2 were statistically significant for all outcomes after Bonferroni correction – the only exception being peritraumatic distress in the persistent group at T0-T1 (Supplementary Table S3). In the persistent trajectory mean scores at T0 and T2 were 10.3 (SD 4.58) and 10.8 (4.43) for anxiety, 11.1 (4.80) and 12.7 (4.96) for depression, 16.9 (7.40) and 18.0 (7.27) for peritraumatic distress, and 38.0 (17.6) and 34.2 (15.8) for well-being (inverted score). The net change between T0 and T2 was statistically significant only for depression and well-being (Supplementary Table S3). The resilient trajectory remained stable over time (e.g., depression 3.39 (2.82) to 3.85 (2.95); well-being 71.6 (14.3) to 69.0 (15.9)). Descriptive statistics for each trajectory are provided in Supplementary Table S2, and the corresponding tests of change over time in Supplementary Table S3.

Multivariable binomial regression analyses (Table 3) showed that being a woman, having an atypical work schedule, working in a COVID-19 unit, and reporting a work overload at T0 were independently associated with the persistent trajectory group across all mental health outcomes. On the other hand, working in medical-technical positions or senior manager roles were protective factors, implying that those in these roles were more likely to be part of the resilient group.

**Table 3:** Factors associated to ‘persistent’ trajectory (poor mental health) versus ‘resilient’ trajectory, *N* = 1,552*.*

	GAD scores			PHQ scores			PDI scores			WHO-5 scores		
Mental health predictors	OR	(95%CI)	*p-value*	OR	(95%CI)	*p-value*	OR	(95%CI)	*p-value*	OR	(95%CI)	*p-value*
Gender												
Man	Ref			Ref			Ref			Ref		
Woman	1.67	(1.23, 2.27)	<.001	1.49	(1.10, 2.05)	0.01	1.59	(1.15, 2.23)	0.01	1.61	(1.23, 2.12)	0.01
Age class												
18–29	1.35	(0.80, 2.25)	0.26	1.62	(0.95, 2.74)	0.07	0.94	(0.53, 1.64)	0.84	1.28	(0.77, 2.11)	0.07
30–39	1.07	(0.76, 1.50)	0.71	1.23	(0.86, 1.74)	0.25	0.98	(0.68, 1.40)	0.90	1.13	(0.82, 1.55)	0.25
40–49	1.01	(0.76, 1.35)	0.93	1.17	(0.87, 1.57)	0.29	0.87	(0.64, 1.19)	0.39	1.23	(0.95, 1.60)	0.29
50–59	Ref			Ref			Ref			Ref		
≥60	0.94	(0.50, 1.70)	0.84	1.45	(0.78, 2.61)	0.23	1.14	(0.59, 2.11)	0.68	0.85	(0.48, 1.49)	0.23
Change of hospital sector since the pandemic												
No	Ref			Ref			Ref			Ref		
Yes	0.99	(0.73, 1.34)	0.95	1.15	(0.85, 1.56)	0.36	0.99	(0.71, 1.36)	0.93	1.21	(0.90, 1.61)	0.36
COVID-19 unit												
No	Ref			Ref			Ref			Ref		
Yes	1.69	(1.13, 2.51)	0.01	1.32	(0.88, 1.98)	0.18	1.48	(0.97, 2.23)	0.07	1.20	(0.81, 1.78)	0.18
Hospital sector												
Women and children	Ref			Ref			Ref					
Surgery	1.28	(0.72, 2.28)	0.4	1.15	(0.64, 2.06)	0.63	1.03	(0.55, 1.91)	0.93	1.21	(0.70, 2.11)	0.63
Intensive care	1.54	(0.93, 2.56)	0.09	1.39	(0.84, 2.32)	0.20	1.41	(0.83, 2.41)	0.21	0.78	(0.48, 1.27)	0.21
Primary care	0.38	(0.18, 0.75)	0.007	0.41	(0.19, 0.81)	0.01	0.39	(0.17, 0.83)	0.02	0.56	(0.31, 0.98)	0.01
Medical specialty	1.17	(0.70, 1.95)	0.55	0.86	(0.50, 1.47)	0.59	1.40	(0.82, 2.41)	0.22	1.10	(0.68, 1.78)	0.59
Diagnostic (radio, labs, pathology)		(0.78, 2.79)	0.22	1.07	(0.55, 2.06)	0.84	1.39	(0.68, 2.79)	0.36	0.90	(0.50, 1.63)	0.84
Readaptation and geriatric	1.16	(0.72, 1.89)	0.54	1.39	(0.86, 2.25)	0.18	1.67	(1.02, 2.77)	0.04	1.31	(0.83, 2.07)	0.18
Neurosciences	0.96	(0.50, 1.81)	0.9	0.89	(0.46, 1.70)	0.73	1.41	(0.72, 2.72)	0.30	1.10	(0.61, 2.00)	0.73
Oncology	0.54	(0.17, 1.43)	0.25	0.20	(0.03, 0.72)	0.03	0.26	(0.04, 0.94)	0.08	0.54	(0.21, 1.26)	0.16
Mental health and psychiatry	0.94	(0.53, 1.65)	0.84	0.68	(0.37, 1.21)	0.19	0.88	(0.48, 1.62)	0.69	0.85	(0.51, 1.42)	0.19
Direction and common service	0.98	(0.57, 1.69)	0.94	1.17	(0.68, 2.03)	0.58	0.98	(0.54, 1.76)	0.94	1.19	(0.72, 1.97)	0.58
Exploitation	1.34	(0.60, 2.94)	0.47	1.02	(0.42, 2.39)	0.96	0.93	(0.36, 2.31)	0.88	0.90	(0.43, 1.87)	0.96
Other	1.09	(0.62, 1.90)	0.77	1.29	(0.73, 2.27)	0.37	1.32	(0.73, 2.38)	0.36	1.02	(0.60, 1.72)	0.37
Hierarchical status												
No manager	Ref			Ref			Ref			Ref		
Middle manager	0.76	(0.53, 1.09)	0.13	1.00	(0.69, 1.43)	0.98	0.54	(0.35, 0.81)	<0.004	0.80	(0.57, 1.11)	0.98
Senior manager	0.58	(0.34, 0.98)	0.04	0.54	(0.30, 0.93)	0.03	0.54	(0.29, 0.96)	0.04	0.66	(0.41, 1.05)	0.03
Teleworking												
No	Ref			Ref			Ref			Ref		
Yes	0.72	(0.49, 1.04)	0.08	0.95	(0.65, 1.38)	0.79	1.04	(0.69, 1.53)	0.87	0.92	(0.66, 1.28)	0.79
Workload												
Idem	Ref			Ref			Ref			Ref		
Underload	0.87	(0.61, 1.22)	0.42	1.02	(0.72, 1.43)	0.92	0.80	(0.55, 1.15)	0.24	0.93	(0.68, 1.26)	0.92
Overload	2.21	(1.69, 2.88)	<0.001	1.68	(1.28, 2.21)	<0.001	1.46	(1.09, 1.93)	0.01	2.00	(1.54, 2.59)	<0.001
Marital status												
In a relationship/Married	Ref			Ref			Ref			Ref		
Single	0.97	(0.69, 1.34)	0.84	1.12	(0.80, 1.57)	0.50	0.98	(0.69, 1.39)	0.91	0.96	(0.70, 1.31)	0.50
Separated	0.80	(0.56, 1.14)	0.21	0.89	(0.62, 1.27)	0.54	0.78	(0.53, 1.14)	0.21	1.14	(0.83, 1.56)	0.54
Widowed	0.44	(0.02, 2.71)	0.45	3.07	(0.64, 16.40)	0.16	0.68	(0.03, 4.55)	0.73	5.44	(0.82, 107)	0.16
Atypical work schedule												
No	Ref			Ref			Ref			Ref		
Yes	1.29	(1.00, 1.67)	0.05	1.33	(1.02, 1.73)	0.03	1.22	(0.92, 1.60)	0.16	1.41	(1.12, 1.79)	0.03
Work contract												
Long-term contract	Ref			Ref			Ref			Ref		
Fixed-term contract	1.17	(0.72, 1.97)	0.53	1.55	(0.92, 2.70)	0.11	1.36	(0.80, 2.42)	0.27	1.10	(0.70, 1.74)	0.11
Student trainee	1.65	(0.36, 7.08)	0.5	1.35	(0.25, 6.05)	0.70	2.54	(0.45, 12.56)	0.26	1.92	(0.41, 10.5)	0.70
Professional categories												
Nurses-Caregivers	Ref			Ref			Ref			Ref		
Physicians	0.81	(0.50, 1.30)	0.39	0.80	(0.48, 1.30)	0.37	0.86	(0.51, 1.43)	0.56	0.87	(0.56, 1.34)	0.36
Administrative Staff	0.97	(0.66, 1.41)	0.87	0.83	(0.56, 1.23)	0.70	0.71	(0.47, 1.07)	0.10	0.74	(0.52, 1.05)	0.70
Logistics staff	0.90	(0.43, 1.86)	0.78	0.47	(0.19, 1.05)	0.08	0.50	(0.19, 1.20)	0.13	0.71	(0.36, 1.42)	0.07
Medical-technical staff	0.59	(0.38, 0.90)	0.02	0.78	(0.51, 1.18)	0.24	0.43	(0.27, 0.69)	<0.001	1.01	(0.69, 1.46)	0.24
Other	0.73	(0.39, 1.32)	0.31	0.45	(0.22, 0.85)	0.02	0.62	(0.31, 1.17)	0.15	0.48	(0.27, 0.83)	0.02

## Discussion

### Distinct mental health trajectories among HCWs (research question 1)

This study reveals the diverse mental health reactions of healthcare workers during the initial two waves of the COVID-19 pandemic. Using a longitudinal design, we identified, for each outcome separately, two trajectories – a ‘resilient’ trajectory with consistently low symptoms and a ‘persistent’ trajectory with consistently elevated symptoms (or, for the WHO-5, consistently low well-being). Because the analyses were outcome-specific, a given worker could follow a persistent trajectory for one outcome and a resilient trajectory for another; only about one in seven followed a persistent trajectory on all four outcomes (Supplementary Table S4). The predominance of resilience in our sample, more than two-thirds for anxiety, depression, and peritraumatic stress, is reassuring, and aligns with prior international research on psychological adaptation during crises (26–29). Nevertheless, a persistent subgroup of 30% participants that remains present does raise concern. These individuals experienced only partial and transient relief between the pandemic peaks, with a significant net worsening over the study period for depression and well-being. Their constant distress displays the limitations of population-level narratives of recovery and reinforces the significance of identifying and supporting vulnerable groups.

A U-shaped symptom curve was observed in both the groups showing higher scores during the two pandemic peaks and improvement in the summer between the waves. The timing and the scores are clearly influenced by the increase in external factors like infection rates, healthcare system strain, and socio-cultural restrictions. Furthermore, the evidence that many HCWs in the persistent group did not return to baseline even during the low-pressure period indicates the inability of some workers to recover spontaneously, quite possibly due to a chronic overburden or lack of structural support (30–35). We thus observe that resilience is not merely an individual trait but a phenomenon shaped by institutional and social conditions.

Our findings are consistent with well-documented disparities within healthcare systems. In our sample, women, nurses, non-managerial staff, and frontline COVID-19 workers were more likely to belong to the persistent trajectory group, underscoring the association between gender, occupational role, and hierarchy and psychological vulnerability. Because no pre-pandemic data were collected, we cannot determine whether the pandemic created or widened these disparities. Our study emphasizes the fact that mental health is not just defined by personal coping styles and exposure levels, and the importance of a structural approach to risk which englobes policies, workplace design, social systems, economic conditions, organizational structures, and inequalities. This involves recognizing the inherent inequities in working conditions, recognition, and decision-making power within hospital systems (33, 35, 36)

### A structural gradient of vulnerability (research questions 2 and 3)

Understanding the risk factors associated with long-term psychological distress identifies the influence of organisational structure and social influence on mental health during a pandemic or crisis. Factors such as irregular work schedules, working in COVID-19 units, feeling overwhelmed with work, and being female are consistently related to declining mental health outcomes. Our study reaffirms that the above components influence mental well-being across the study period and not just short-term stress. These effects last between the different pandemic waves, indicating that these risks have short and long-term persisting effects. Crucially, our data point to a professional and hierarchical gradient in vulnerability. Nurses and caregivers, who essentially have physical and emotional demanding role close to the patient, were excessively represented in the group that had persistent symptoms. These workers are frequently confronted with high workloads, irregular hours, limited opportunities for advancement, and low recognition (37, 38). While managing their family and personal responsibilities, their professional challenges were exacerbated by an increase in patient load, a shortage of staff, and a high risk of exposure, along with their other personal responsibilities. These cumulative pressures may have declined their ability to recover, especially in the absence of institutional support (30, 34, 39).

A higher resilience was observed in individuals in managerial or medical-technical roles, implying that having the possibility of autonomy and decision-making authority in an institution could be a protective factor. Our findings reflect Karasek and Theorell's job demand-control model (36), which implies that workers with higher control over their tasks are better equipped to cope with stress. For HCWs, managerial positions provide access to key resources like flexible schedules, following work progress, peer appreciation, and strategic vision. These can help reduce psychological overload.

The determinants of trajectory membership were not uniform across outcomes. Being a woman, reporting work overload, and holding a senior management position (protective) were associated with trajectory membership for all four outcomes, whereas other associations were outcome-specific: working in a COVID-19 unit was associated with the persistent trajectory for anxiety only, medical-technical positions were protective for anxiety and peritraumatic distress but not for depression or well-being, and an atypical schedule was associated with anxiety, depression and well-being but not with peritraumatic distress. Frontline exposure was thus associated primarily with anxiety, whereas workload and gender were associated with distress across all dimensions – a distinction of practical relevance when targeting preventive measures.

We identified that personal toughness or professional superiority does not necessarily define the resilience gradient. Resilience in this crisis is clearly influenced by the degree of exposure and protection, depending on the work role and organizational structure. The level of resilience or vulnerability of an individual was shown to be defined both by their capacity to adapt with the systemic support they received. While the association between heavy workloads and poorer mental health is well established, our longitudinal trajectories add that vulnerability is structurally patterned by occupational role and hierarchy and persists across waves. This systemic view aligns with the resilient-healthcare literature (e.g. (40),. This supports complementing individual resilience programmes with organizational measures that target the working conditions generating fragility.

### Modifiable workplace drivers of distress (research question 2)

We observed that the mental health pathway of healthcare workers was impacted by their occupational ecosystem. Irregular schedules and work overload were associated with a higher presence of persistent symptoms. These factors were not random or incidental; they illustrate intrinsic issues that pre-existed in the human resources management and were exacerbated during the pandemic. Illness-related absences, sudden urgent assignments, and workforce shortage had an impact on adequate staffing and maintaining a constant workflow. This not only translated into an explosion of workloads but also hampered recovery, especially for workers with limited control over their shifts. The relationship between workload and poor mental health outcomes is well-documented (31, 41–45), and our longitudinal data show how such exposures were associated with sustained symptoms across the first two waves of the pandemic. The presence of lasting mental health distress despite the temporary relief between pandemic waves suggests that once a threshold of overload is crossed, the probability of recovery decreases without deliberate organizational intervention. Psychological vulnerability is significantly affected by irregular work timings, rotating and night shifts, which disturb circadian rhythms, sleep quality and hamper sleep quality (44, 45).

Workers in frontline COVID-19 unit settings demonstrated higher levels of anxiety and distress in both Swiss and international contexts (30, 46, 47). We identified early frontline deployment in these units as a key risk factor as well. These frontline roles higher probability of psychological insecurity and vulnerability due to frequent exposure to professional ethical conflicts, challenging team dynamics and structure, and possible perception of degradation in patient care. Some studies have suggested that being considered a “key worker” may offer some protection for certain outcomes, such as depression (48); although in reality, it remains context-dependent and surpassed by the concentration of workload and pressure in high-risk settings.

Together, these findings emphasize the need to shift towards proactive intrinsic organizational mental health care in preference to reactive mental health support (33, 34, 49). These outcome-specific results have practical implications for occupational health. About one in seven workers followed a persistent trajectory across all four dimensions and would be flagged by any single screen, whereas nearly half were resilient throughout; between these, a substantial group was vulnerable on specific dimensions only, so screening limited to a single instrument would miss part of the at-risk workforce. Because persistently low well-being captured most workers with persistent symptoms while also identifying a broader group, the WHO-5 may serve as an efficient first-line screen, with dimension-specific instruments used to target support.

Institutions must recognize that poor psychological health in workers is not simply a subsequent effect of crisis exposure. It is a mirror of the organization's ability to manage (or fail to manage) uncertainty, pressure, and resource constraints (30, 34, 50). Our study suggests that long-term mental healthcare of healthcare personnel depends significantly on the occupational ecosystem they are embedded. The importance of their internal resilience and capacity to deal with adverse situations is an undeniable resource, but there are many other work-related factors to account for.

It has become imperative to redefine resilience, broadening its definition beyond psychological traits to encompass institutional design (34, 40). Can resilient healthcare systems then be defined as those that expose workers to risk more equitably, provide proactive support to frontline workers, and have the capacity to be adaptable to crisis without sacrificing the well-being of their staff?

Our study has some limitations. Outcomes were self-reported, and the 25% baseline response rate and ∼55% attrition may have introduced selection bias. We relied on general distress measures (GAD-7, PHQ-9) rather than work-specific instruments such as burnout, so we cannot quantify the share of distress attributable to work as opposed to the broader pandemic context. Physicians were under-represented (11% of participants), although they are among the most exposed groups, which may limit representativeness for that profession. The study was conducted in a single tertiary hospital, and the PDI was used beyond its validated immediate-trauma window. The online data collection method may have introduced bias in the representativeness of certain professionals. For instance, non-clinical staff, such as logistical, cleaning, and catering staff, may have had limited computer access. As is common in online surveys, older participants may be overrepresented. Also, the absence of pre-pandemic mental health data is a limitation: our longitudinal design documents change across the pandemic waves but cannot establish a pre-pandemic baseline.

Nevertheless, it is essential to recognize that these points could influence the interpretation of our findings. The main strength of our study lies in its large sample size and longitudinal design. It provides a valuable exploration of the complexity of mental health trajectories and their interrelationship with socio-demographic and organisational variables. Our study contributes to a more nuanced understanding of the pandemic's impact on mental health.

## Conclusion

In this single-centre longitudinal study, roughly one third of healthcare workers followed a persistent distress trajectory across the first two waves of the COVID-19 pandemic, and membership in that trajectory was associated with being a woman, working in a COVID-19 unit, reporting work overload, and having an atypical schedule. These results support viewing the psychological toll of a crisis as partly a system-level outcome rather than a matter of individual fortitude alone: healthcare systems should address the working conditions associated with persistent distress in order to retain their workforce and maintain quality of care in future crises.

Our findings also point to the value of longitudinal monitoring rather than one-off screening. Because a substantial subgroup did not recover between the two waves, single time-point assessments and single-session debriefings are likely to miss delayed or non-recovering profiles. Tracking trajectories over time – ideally through occupational health services and supported by organizational leadership – would help identify these workers and target support.

More broadly, the trajectories identified here can serve both as a diagnostic tool, by revealing which groups are most vulnerable and under which conditions, and as a basis for organizational measures addressing workload, schedule regularity, equity, and recognition. Future research should test such organizational interventions prospectively, incorporate work-specific measures (e.g., burnout) and a pre-pandemic baseline, and extend follow-up beyond the acute phases of a crisis.

## Data Availability

The raw data supporting the conclusions of this article will be made available by the authors, without undue reservation.
