# Supplementary material for: Trajectories of mental health and well-being across healthcare workers during COVID-19 pandemic: a Swiss longitudinal study
Source: Front Health Serv. 2026 Oct 27;6:1883340. doi: 10.3389/frhs.2026.1883340 (PMC13747960; doi:10.3389/frhs.2026.1883340)
Supplement: Supplementary file 1 [file Datasheet1.pdf]

Supplementary Figure S1 - *Cluster-number selection and trajectory solutions*

For each outcome (GAD-7, PHQ-9, PDI, WHO-5), longitudinal k-means clustering (kml package, R) was estimated for two to seven clusters with ten re-initializations each. The number of clusters was selected using the quality criteria provided by kml, primarily the Calinski-Harabasz index. For all four outcomes the two-cluster solution maximized the Calinski-Harabasz criterion and was stable across all ten re-initializations, distinguishing a lower-symptom ("resilient") from a higher-symptom ("persistent") trajectory. In each panel below, the left plot shows the Calinski-Harabasz criterion obtained for each candidate solution across the ten re-initializations (the line labelled "2" is highest and flat), and the right plot shows the resulting two-cluster trajectories with the proportion of participants in each group.

A – Anxiety (GAD-7)

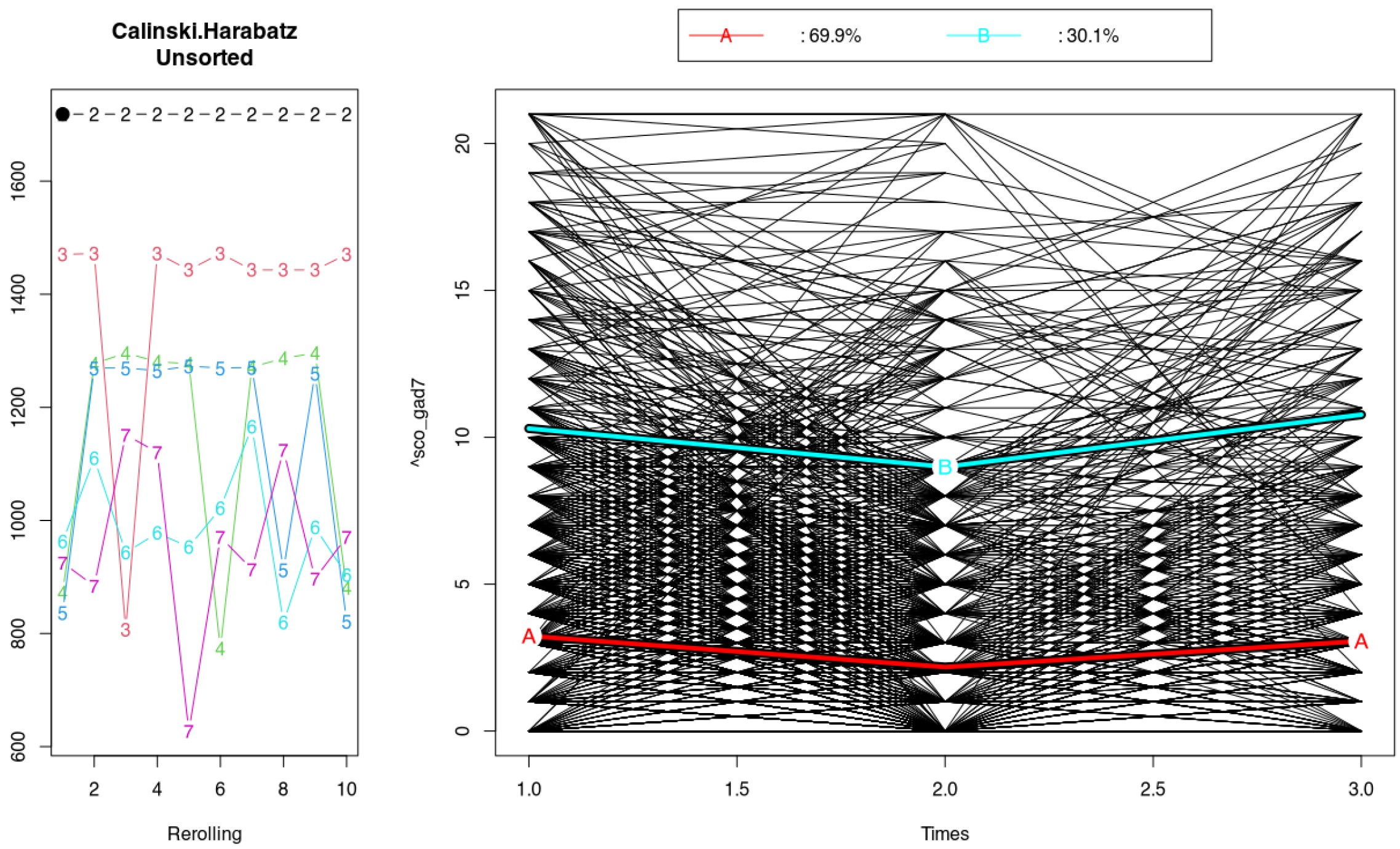

Figure S1A. *Cluster-selection criterion (left) and the selected two-cluster trajectory solution (right) for anxiety (GAD-7). Resilient: 69.9%; persistent: 30.1%.*

B – Depression (PHQ-9)

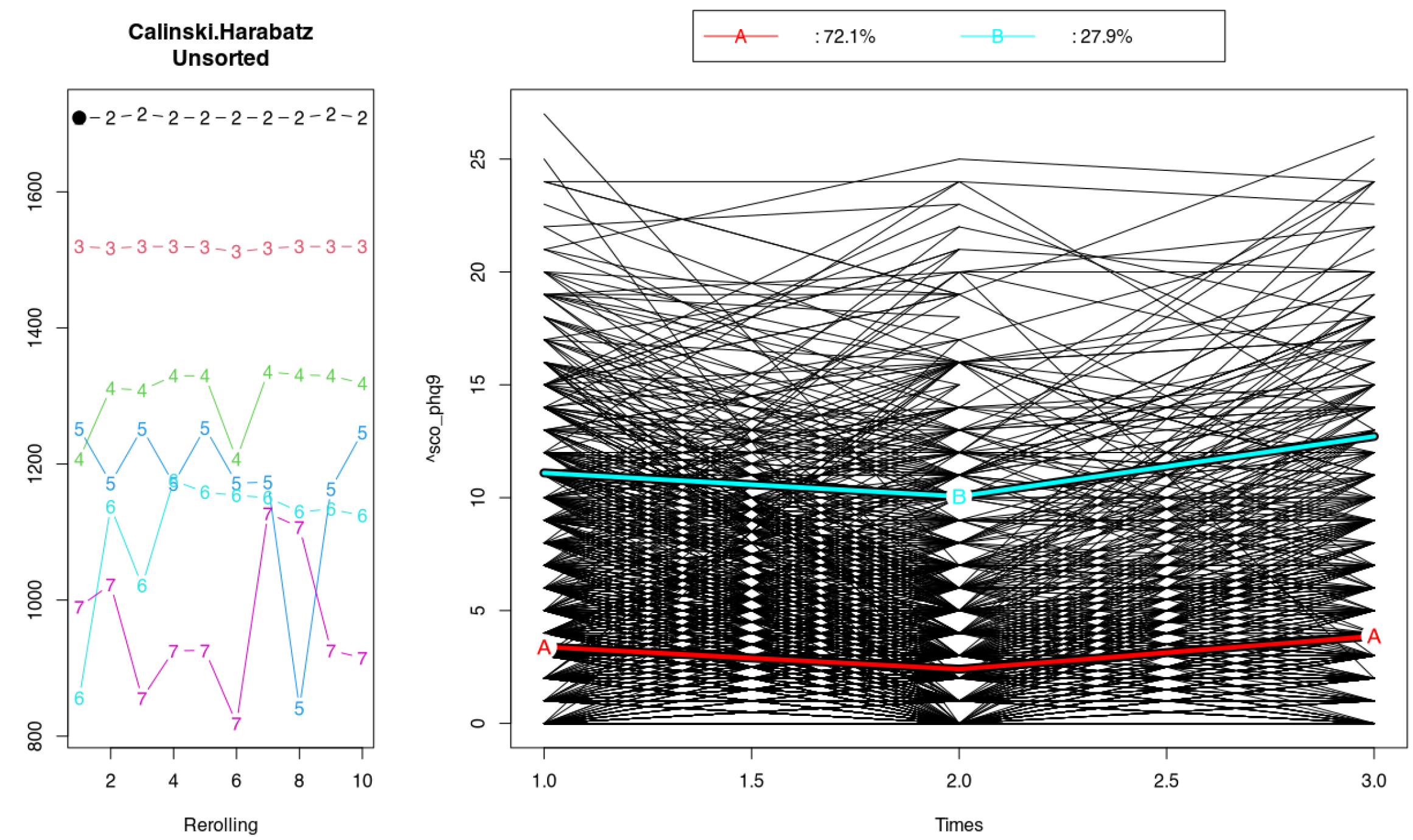

Figure S1B. *Cluster-selection criterion (left) and the selected two-cluster trajectory solution (right) for depression (PHQ-9). Resilient: 72.1%; persistent: 27.9%.*

C – Peritraumatic distress (PDI)

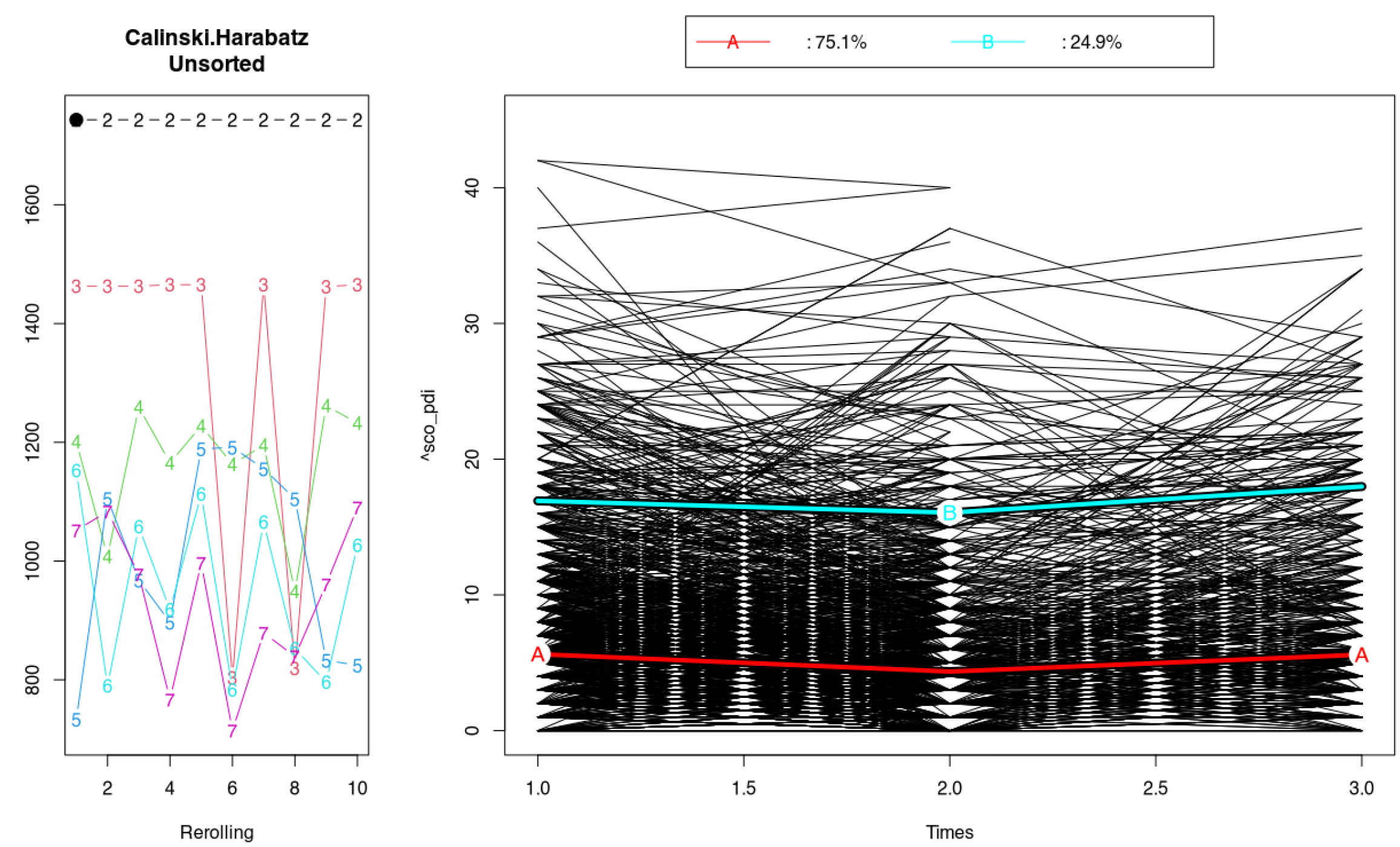

Figure S1C. *Cluster-selection criterion (left) and the selected two-cluster trajectory solution (right) for peritraumatic distress (PDI). Resilient: 75.1%; persistent: 24.9%.*

D – Well-being (WHO-5)

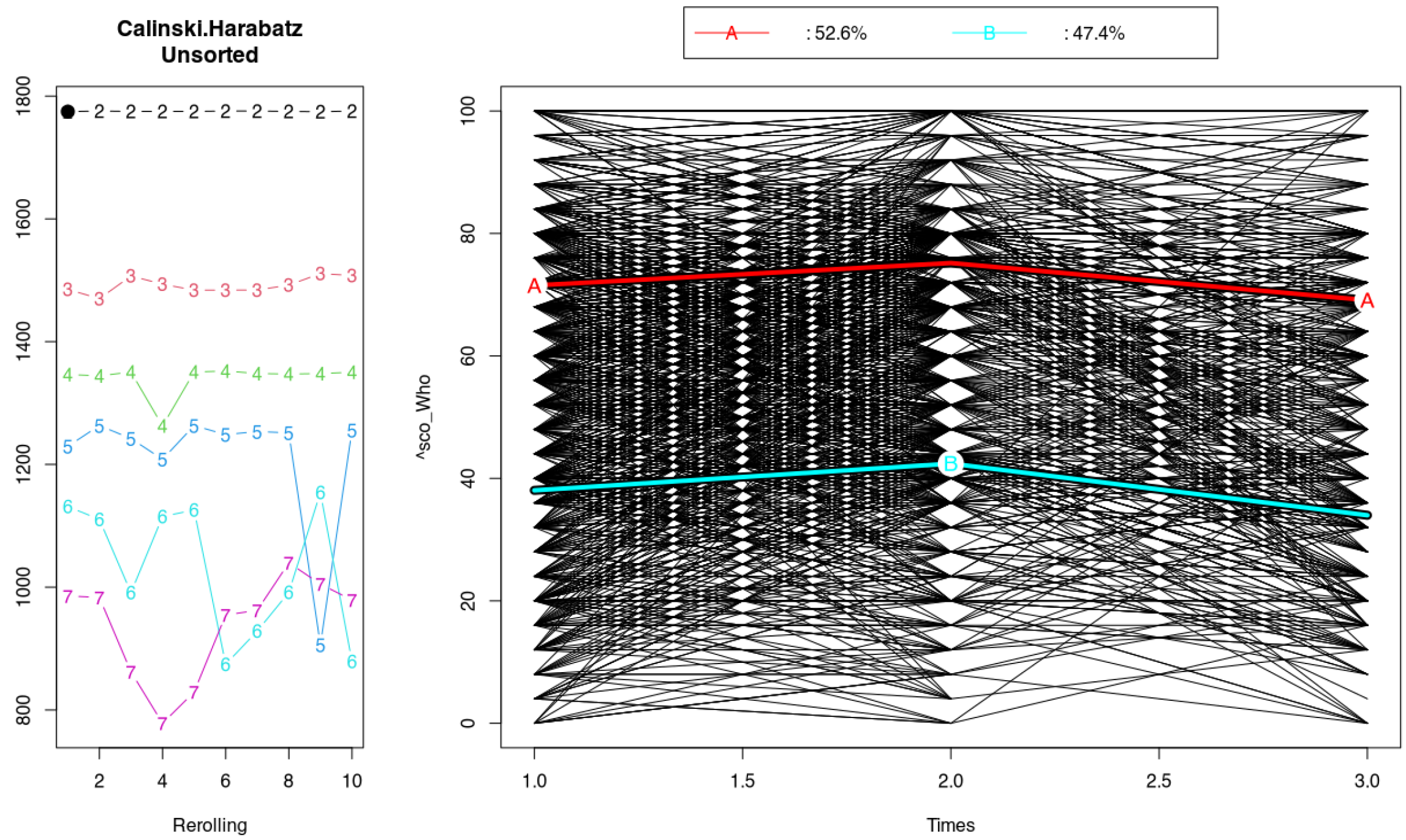

Figure S1D. *Cluster-selection criterion (left) and the selected two-cluster trajectory solution (right) for well-being (WHO-5). For the WHO-5 a higher score denotes better well-being, so the upper trajectory is the resilient group. Resilient: 52.6%; persistent: 47.4%.*

Table S1

Comparison between participants and studied population's characteristics

Characteristics	Population (N=13,570)	Participated in follow-ups (N=1,552)
	***N* (%)**	***N* (%)**
**Demographics**		
**Age class**		
18–29	1561 (11)	116 (7)
30–39	3569 (26)	315 (20)
40–49	3540 (26)	522 (34)
50–59	3627 (27)	531 (34)
60+	1293 (10)	68 (5)
**Gender**		
Man	4350 (32)	384 (25)
Woman	9220 (68)	1165 (75)
N/A	0 (0)	3 (0)
**Professional categories 1**		
Nurse / caregivers	5661 (42)	700 (45)
Administrative staff	2145 (16)	307 (20)
Physician	2948 (17)	177 (11)
Medical-technical staff	1692 (12)	200 (13)
Logistics staff	1704 (13)	91 (6)
Others	0 (0)	77 (5)
N/A	0 (0)	0 (0)
**Hospital sector**		
Surgery	845 (6)	95 (6)
Intensive care	1177 (9)	162 (10)
Primary care	651 (5)	90 (6)
Medical speciality	1171 (9)	145 (9)
Women and children	1651 (12)	155 (10)
Diagnostic (radio, lab, pathology)	871 (6)	95 (6)
Readaptation and geriatry	1056 (11)	184 (12)
Neurosciences	657 (5)	74 (5)
Oncology	321 (2)	31 (2)
Mental health and psychiatry	992 (7)	123 (8)
Direction and common service	2173 (16)	187 (12)
Exploitation	1517 (11)	90 (6)
Other	38 (0)	121 (8)

Supplementary File S2 - *Descriptive statistics of the mental-health and well-being trajectories*

Trajectory	May 2020 (T0)	Sept. 2020 (T1)	Dec. 2020 (T2)
**Anxiety — GAD-7 (range 0–21)**			
**Resilient**	3.24 (2.55)	2.18 (2.16)	3.06 (2.57)
**Persistent**	10.3 (4.58)	8.99 (4.69)	10.8 (4.43)
**Depression — PHQ-9 (range 0–27)**			
**Resilient**	3.39 (2.82)	2.40 (2.17)	3.87 (2.95)
**Persistent**	11.1 (4.80)	10.1 (5.22)	12.7 (4.96)
**Peritraumatic distress — PDI (range 0–52)**			
**Resilient**	5.64 (3.78)	4.35 (3.25)	5.60 (3.94)
**Persistent**	16.9 (7.40)	16.1 (7.54)	18.0 (7.27)
**Well-being — WHO-5 (range 0–100; higher = better)**			
**Resilient**	71.6 (14.3)	75.2 (13.2)	69.0 (15.9)
**Persistent**	38.0 (17.6)	42.5 (18.4)	34.2 (15.8)

Note. Values are mean (standard deviation) of the observed scores at each assessment, by trajectory group, for participants assigned to a trajectory (N = 1,552). Trajectories were derived by univariate longitudinal k-means clustering of each outcome. Higher scores indicate more symptoms for the GAD-7, PHQ-9 and PDI, and better well-being for the WHO-5. For all four outcomes, scores in both groups followed a U-shaped pattern, improving between the two waves (T1) and returning toward or above baseline at the second wave (T2).

Supplementary File S3 - *Tests of change over time within each trajectory*

Trajectory	T0 – T1	T1 – T2	T0 – T2
**Anxiety – GAD-7**			
**Resilient**	**< 0.001**	**< 0.001**	0.048
**Persistent**	**< 0.001**	**< 0.001**	0.027
**Depression – PHQ-9**			
**Resilient**	**< 0.001**	**< 0.001**	**0.013**
**Persistent**	**< 0.001**	**< 0.001**	**< 0.001**
**Peritraumatic distress – PDI**			
**Resilient**	**< 0.001**	**< 0.001**	0.526
**Persistent**	0.030	**< 0.001**	0.027
**Well-being – WHO-5**			
**Resilient**	**< 0.001**	**< 0.001**	**0.007**
**Persistent**	**< 0.001**	**< 0.001**	**< 0.001**

Note. For each outcome and trajectory group, scores were compared between assessments (T0 = May 2020, T1 = September 2020, T2 = December 2020) using paired t-tests. The three comparisons within each outcome were corrected for multiple testing with a Bonferroni-adjusted threshold (corrected α = 0.0167). Values are p-values; those significant after correction are shown in bold. The consecutive changes (T0–T1 and T1–T2) were significant for almost all outcomes in both groups, whereas several overall changes (T0–T2) were not significant after correction (GAD-7 and PDI in both groups). This pattern is consistent with the U-shaped course of symptoms, with improvement between the two waves followed by a return toward baseline at the second wave.

Supplementary Table S3 – Concordance between the four outcome-specific trajectory classifications
Cluster analyses were conducted separately for each outcome (GAD-7, PHQ-9, PDI, WHO-5). This table describes the overlap between the resulting classifications for the 1527 participants classified on all four outcomes. Persistent trajectories comprised 29.9% of participants for anxiety, 27.6% for depression, 24.8% for peritraumatic distress and 47.5% for well-being.

Panel A. Number of outcomes on which a participant followed the persistent trajectory

Number of outcomes with a persistent trajectory	n	%
0 (resilient on all four)	706	46.2
1	261	17.1
2	184	12.0
3	152	10.0
4 (persistent on all four)	224	14.7
**Total classified on all four outcomes**	**1527**	**100.0**

Panel B. Pairwise agreement between classifications

Outcome pair	Agreement (%)	Cohen's κ	Both persistent (n)
GAD-7 vs PHQ-9	84.1	**0.61**	317
GAD-7 vs PDI	83.7	**0.59**	293
GAD-7 vs WHO-5	73.3	**0.46**	387
PHQ-9 vs PDI	83.1	**0.56**	271
PHQ-9 vs WHO-5	74.9	**0.49**	381
PDI vs WHO-5	71.6	**0.42**	335

Note. *A participant may follow a persistent trajectory for one outcome and a resilient trajectory for another; membership is therefore outcome-specific. Agreement among the three symptom scales was moderate to substantial (κ = 0.56–0.61), whereas well-being (WHO-5) defined a broader persistent group with weaker agreement with the symptom scales (κ = 0.42–0.49): 85–90% of workers on a persistent symptom trajectory also reported persistently low well-being, but the converse was less frequent (46–53%).*
